# Magnetorotational instability in dense electron–positron–ion plasmas

**DOI:** 10.1038/s41598-023-42397-3

**Published:** 2023-09-15

**Authors:** S. Usman, A. Mushtaq

**Affiliations:** 1 Department of Physics, University of Wah, Wah Cantt, 47010 Pakistan; 2 Department of Physics, FBAS, International Islamic University (IIUI), Islamabad, 44000 Pakistan

**Keywords:** Astronomy and astrophysics, Astronomy and planetary science, Physics

## Abstract

We in this manuscript analyzed the magnetorotational instability (MRI) by using a multi-component quantum fluid model with the effect of spin magnetization in a differentially rotating degenerate electron–positron–ion (e–p–i) quantum plasma. The electrons and positron having the same mass but opposite charge are taken to be degenerate whereas ions are considered as classical owing to their large inertia. The general dispersion relation is derived and a local dispersion relation for MRI is obtained by applying MHD approximations. To obtained MRI and to analyze the results numerically, reduced dispersion relation is derived using the local approximations. The obtained results are applied to the astrophysical situations exist there in the interiors of White Dwarfs and neutron stars. Contribution from spin magnetization and the number densities of electrons and positrons plays a vital role in the dynamics and can alter the instability. The increase in the electron number density, hence spin magnetization enhances the growth rate of the mode and leads the system to instability which results in the core collapse of certain massive stars.

## Introduction

In degenerate plasmas, the electrons are closely packed together with the maximum allowed density by quantum mechanics at a given pressure. The quantum effects in such a situation play a vital role when the charged particles de-Broglie wavelength $\lambda _{De}(=\hslash /m_{e}v_{te})$ stands comparable to the scale length of the system e.g. interparticle distances $n^{-1/3}$, where $\hslash ,$ $m_{e}$, *n*, and $v_{te}$ are the reduced planck’s constant, mass of electron, equilibrium particle density and particle thermal speed respectively. These type of plasmas can be found in the interior of densest astrophysical object^1^ (degenerate stars). There are mainly three classes of the degenerate stars known as white dwarfs (WDs), neutron stars (NS), and black holes (BH). The WDs are supported against the collapse by the electron degeneracy pressure, while the neutron stars are largely supported by neutron degeneracy. Black holes are the completely collapsed stars as they collapsed to a singularity. It is worthy to notice that the properties of a quantum plasma present in the interiors and surrounding of these degenerate stars, alters significantly from a classical plasma.

There has been an increasing interest in describing collective quantum effects in plasmas using quantum fluid theory (QFT). The regime of interest is, when the de Broglie wavelength of the charge carriers is comparable to the dimensions of the system, then quantum mechanical diffusion and tunneling cannot be ignored. This effect is described in QFT through the Bohm potential. The Bohm potential first appeared in Madelung’s 1926 alternative to the Schrödinger equation^2^ and has been re-derived in various ways^3^ in QFT, the intrinsically quantum term is the Bohm potential, which describes quantum mechanical diffusion or tunneling. Motivated by application to solid state plasmas, the response tensor for a completely degenerate nonrelativistic electrons gas has been known since the 1950s^4^. The generalization to a fully relativistic quantum treatment for electrons and positrons, including the nonlinear response tensors, as well as the linear response tensor is available, and has been called quantum plasmadynamics (QPD)^5^. All relativistic quantum effects are included in QPD (like degeneracy, quantum recoil, spin, etc.). QPD is a more rigorous theory than QFT, and all the results of QFT should be derivable from QPD. However, the relationship between these two approaches is not immediately obvious. A notable difference in comparing them is that QFT is formulated in coordinate space (t,x), whereas QPD is formulated in Fourier space (ω,k). Quantum mechanical diffusion or tunneling does not appear explicitly in a Fourier space formulation, but it must be included in the known quantum effects.

Since the statistical description changes from Maxwell Boltzmann’s to the Fermi Dirac, many attempts were made^6–8^ to develop a quantum fluid theory. To address the situation of degenerate plasma regimes, quantum magnetohydrodynamic (QMHD) model was formulated by Haas in 2005^9^. The QHD equations are derivable from the electromagnetic Wigner equation^10^ by taking the relevant moments of the distribution function, one can find out both the linear and nonlinear plasma responses to electromagnetic fields, which constitutes the primary objective of plasma description^11^. The necessary thing for the Quantum hdrodnamics (QHD) equations is that all ensemble averages can be replaced by the mean quantities. However, a model whose validity rests on several assumptions, imposing important limitations on the model. As plasma constitutes mainly of electrons and ions, only the electrons exhibit quantum degeneracy, so the limitations can only apply exclusively to the electron-component. It gives rather semi-classical picture of the system. For the plasma to be considered ideal (weakly coupled), all types of interactions should be significantly weaker than the quantum kinetic energy $(\Gamma _{q}<<1)$. The interaction of the particles is approximated using the mean field approach. The QHD equations are applicable to large resolvable length scales that means they are suitable for the long wavelength limit $(\lambda>>\lambda _{TF})$. Notably, the energy transport equation and exchange interactions are ignored in this context, which can be addressed by considering the second-order moment of the Wigner function equation to address the model correctly^12^. Under the stated hydrodynamic approximations, it is feasible to analyze the characteristics of a quantum electron gas with higher precision. This can offers an easy way to explore linear waves and instabilities, offering valuable insights into the significant impact of quantum effects in some denser plasma regimes that are usually found in astrophysical environments.

Afterwards the QHD model was extended to spin quantum plasmas by Brodin and Marklund^13^, which is another effect in addition to Bohm potential that can be included to the dynamics of classical plasmas, for instance the possibility for large-scale magnetization. The spin statistics using Pauli spin matrices for many particle systems has been well explained^14, 15^. Using the magnetization theory Langvin (classical interpretation using Maxwell–Boltzmann distribution), and then Pauli (quantum mechanical interpretation using Fermi–Dirac distribution) explained the paramagnetic behavior of the plasma. Based on the density matrix, other effects relevant to spin has been well explained by the density functional theory such as exchange-correlation effect due to spin-up and spin-down electrons of many particle quantum hydrodynamics^16, 17^. Various approaches have been adopted to derive QHD equations^18–20^ with its applications to quantum plasmas involving spinning particles. These equations incorporate important variables such as the particle concentration, the momentum density, velocity field and the distribution function, which characterizes all particles of a given species irrespective of their spin directions. For more details see also^21–23^. To account for the difference in the number of particles in different spin states, these models include the spin density ${\textbf{S}}$ or the magnetization ${\textbf{M}}$. However, it is noteworthy that these models do not explicitly distinguish between the spin-up and spin-down states of individual particles.

The applicability and relevance of this model to the solid state plasma, dusty plasma and dense astrophysical plasma were discussed to investigate the properties of hydrodynamic waves and instabilities^24, 25^. Using the non-relativistic Pauli equation for spin-1/2 fermions (electrons), Brodin and Marklund used spin magnetohydrodynamics (MHD) to formulate the governing dynamics of spin quantum plasmas^26^. It was stated that, spin effect is of great importance in a strongly magnetized, low temperature and high density plasma i.e degenerate stars. They (Brodin and Marklund^13^) developed the theory of spin MHD by neglecting the contribution from the off-diagonal part of spin stress tensor^27^. In case of many-particle systems spin evolution term $$(S_{m})$$ does not contain complete information of spin. Our spin MHD model is based on an approximation, which will be justified when the contributions from the off-diagonal components of spin (interference of different spins) are absorbed in the many-body effects of the spin stress tensor. Another limitation to the proposed model is, it can only be applied to colder plasmas within the density range $$(10^{30}$$–$$10^{40}$$ cm$$^{-3})$$. Moreover, a quantum approach based on Fermi liquid or similar theories based on thomas fermi theory would seem to be the most promising approach to describe spin quantum plasmas^28^. It is possible that SQHD could be re-formulated and new predictions from it tested against experiments in the appropriate regimes^29^.

Beyond these limitations, Misra et al.^30^ studied the influence of the intrinsic spin of electrons on the propagation of circularly polarized waves in a magnetized plasma. Safdar et al.^31^ investigated magnetosonic waves in the presence of degenerate pressure due to Landau diamagnetic levels and Pauli spin magnetization and explored a new propagation mode. A model for dense degenerate plasmas that incorporates electron spin^32^, magnetosonic solitary waves^33^, effects of the spin on the EM wave modes in magnetized plasmas^34^, basic properties of magnetosonic waves in a magnetorotating spin quantum plasma^35^ and instability of Terahertz (THz) plasma waves^36^ in quantum field effect transistors (FETs) with the spin effects are extensively studied and were found to play major roles in specifying the nature, structures and features of astrophysical (neutron stars and white dwarfs) and laboratory (semiconductor) plasmas.

The effect of strong *B* field has many applications in an astrophysical surrounding such as pulsars^37^ and magnetars^38^. Haas and Mahmood^39^ studied the Nonlinear ion-acoustic solitons in a magnetized quantum plasma with arbitrary degeneracy of electrons and the results are validated by comparison with the quantum hydrodynamic model including electron inertia and magnetization effects. Asenjo et al. developed a hydrodynamical^40^ and kinetic model^41^ for relativistic and semirelativistic spin quantum plasmas. Therefore alot of focus has been given to these extreme environments especially in the regimes of strongly magnetized plasmas. In dense astrophysical regimes, such as the atmospheres of massive WDs and the interiors of NS, the quantum corrections to MHD can be very important.

In comparison to the pure electron–positron (e–p) plasma, the conventional electron–ion (e–i) plasmas behave in a different way, because in the former, the electrons–positrons plasma represents a class of equal mass and opposite charge. Such a pair plasmas are believed to exist in the high energy environments, from the first few seconds of the Big Bang. Consequently, the positron concentration strongly alters the wave properties of the electrostatic and electromagnetic modes in e–p plasmas. The positron presence in such a multi-species plasma in these dense environments has been confirmed in a various number of experiments and environments^42, 43^ e.g., in the polar regions of NS, in the active galactic nuclei (AGN), in the solar flares^44^, at the centre of our own galaxy^45^ and in pulsar magnetosphere^46^. The creation of positrons is due to the interactions of intense pulses of laser with plasma^45^ and also due to the collapse of WDs to NS and also observed in radio emission from pulsar magnetosphere^47, 48^, Jet compositions (pair electron–positron plasma), by detecting radio emission from quasars using VLBA^49^ and in Payload for Antimatter Matter Exploration and Light-nuclei Astrophysics (PAMELA) experiments^50^ and by the process of neutronization and by thermal emission^51^. For more details experimental observations see^52, 53^.

In addition to the electrons and positrons, a small fraction of ions has also been observed in the recent observation by the advanced satellites for astrophysics and cosmology^54^, the plasma is an admixture of electrons, positrons and ions. With the introduction of ions as an ingredient to the usual e–p plasma the response of the plasma greatly change. The positrons have enough lifetime that the normal two-species (e–p) plasma becomes a three-species electron–positron–ion (e–p–i) plasma. Naturally, positrons are an ingredient that is present everywhere in compact astrophysical objects and is therefore, the existence of dense e–p plasmas are expected there. A high pair annihilation rate due to a very large densities of electrons and positrons is expected there in these dense objects. However, some investigations^55, 56^ have been made for some density ranges concerning WDs, where the rates of annihilation can be ignored and positrons have enough much lifetime to contribute in collective plasma phenomena^57^. Due to unit mass ratio, in many respects e–p plasma behave in a different way from usual e–i plasmas. This feature makes the investigation of e–p plasma extremely worthy, both for the fundamental physics and for astrophysical interests. The presence of positron in these astrophysical surroundings is certain, especially regarding electron–positron release phenomena via the neutrino annihilations and neutrino absorptions on to the nuclei. Till now, in study of MRI instability the role of positron has usually been neglected. In order to understand the dynamics, it is important to study the state of such a plasma and the dynamics of these species by employing a configuration of rotating object and suitable non-relativistic model (fluid model).

In the recent past decades a number of theories to describe quantum plasma and hydrodynamic stability in magnetized plasmas are developed with its importance to astrophysical environments^58–60^. One of the different instabilities arise in rotating astrophysical dense object is Magnetorotational Instability (MRI). It is a type of MHD instability initially addressed by Velikhov^61^ in 1959 and then after confirmed by Chandrasekhar^62^ in 1960 while studying the Taylor Couttee flow in the concentric differentially rotating cylinders. For almost three decades MRI is out of the context from the main stream research until Balbus and Hawley^63^ in 1991 applied the concept to the accretion disks around a massive central objects. They showed that the growth rate of the MR instability is independent of the magnetic field strength, even a low magnetic field can change the stability of the system. These disks are stable hydrodynamically but they are unstable magnetohydrodynamically, leading to the disk turbulence and transport of angular momentum^64–66^. MRI is also expected to act as a dynamo in the accretion disks^67^. Hereinafter, there is a growing interest in MR instability applications concerning the astrophysical problems in various magnetized accretion disks^68–70^. Different models, various analytical explanations and numerical analysis has been performed to explain the dynamics of MRI in different situations i.e., Single^63^ and two fluid model^71^ was developed with effect of magnetized and un-magnetized plasma, The effect of viscosity in rotating plasma^72^ and rotating dusty plasmas including dissipation^73^. The Incompressible magnetohydrodynamics simulations is presented^74^ in spherical geometry with explicit diffusivities where the differential rotation is forced at the outer boundary. More recently, Nonlinear development of MRI in circularly magnetized eccentric disks^75^, impact of the MRI on the evolution of massive stars^76^, smoothed particle magnetohydrodynamics method^77^ with the geometric density average force expression and the mean field dynamo effect on MRI^78^ are extensively presented. The growth rate of MRI in circumstellar disks^79^ is investigated with the effect of changes in the strength and direction of the magnetic field and reported that the MRI active region possibly exists with a weak magnetic field. The vertical shear instability in poorly ionized, and magnetized protoplanetary discs MRI in all three frequency ranges (low, intermediate and high) of weakly ionized electron–ion–neutral (e–i–n) and (e–p–i) plasma has been investigated by using the classical multi-fluid approach^80, 81^. The purpose of this manuscript is to examine the instability in these regimes in a multi-fluid framework under the influence of quantum correction term in the form of spin magnetization force. Other correction terms e.g. relativistic correction terms, Quantum Bohm potential, pressure degeneracy and exchange correlation effect are not yet included in this work and planed to be included in future to develop a full quantum description of MRI mechanism in dense objects.

MRI for now can be considered as an important candidate in the core collapse of degenerate stars and for many other dynamical behaviors. In this work we examined MRI in three species (e–p–i) dense plasma by introducing quantum correction terms to the equation of motion governing the dynamics. Generally, in dense astrophysical objects, ions provides inertia, where the electrons and positrons are considered to follow the electron/positron degeneracy pressure to support them against the gravitational collapse. Solving the QMHD equations coupled to the Maxwell’s equations we derived the generalized dispersion relation. The quantum contribution from the ions are ignored because of its large mass in comparison to the electron and positron. Their quantum behavior depends upon degeneracy parameter which is larger than unity for quantum case. The dispersion relation is limited to certain MHD conditions to obtain a reduced dispersion relation. The electron and positron densities and spin magnetization effects reveals some important consequences on the instability growth rate. We in this work are intended to make a mathematical and numerical investigations of MRI by looking into the quantum viewpoint of dense astrophysical objects.

This manuscript is arranged as, In “Model Equations and dispersion relation”, the basic quantum hydrodynamic equation of motion for e–p–i plasma along with the Maxwell’s equations are presented. Based on the model equations, the dispersion relation for the e–p–i plasma is obtained. In “Reduced dispersion relation”, the reduced dispersion relation is obtained with certain MHD limitations. Section “Results and discussion” contain the detailed devoted results and discussion, and Finally, in “Conclusions”, the conclusions of the work are presented.

## Model equations and dispersion relation

We consider an axisymmetric, collisionless, fully degenerate and quasi-neutral electron–positron–ion (e–p–i) plasma embedded in homogenous external magnetic field ${\textbf{B}}=B{\hat{z}}$. Using the standard cylindrical geometry $(r,\theta ,z),$ the plasma rotates in the azimuthal $\theta$ direction with an angular frequency $\Omega =\Omega (r)$. The equilibrium quantities are respectively given as ${\textbf{B}}_{0}=(0,0,B_{0}),$ ${\textbf{E}}_{0} =(E_{0},0,0)$, ${\textbf{v}}_{j0}=(0$, $r\Omega ,0)$ and $P_{j0}=P_{j0}(r)$. The dynamics of such a system is governed by continuity and multi-fluid hydrodynamic momentum equation^82^ expressed as

1 $$\begin{aligned}{} & {} \frac{\partial }{\partial t}n_{j}+\mathbf {\nabla }\cdot (n_{j}{\textbf{v}}_{j})=0 \end{aligned}$$

2 $$\begin{aligned}{} & {} \rho _{j}\frac{d_{j}}{dt}{\textbf{v}}_{j}+\mathbf {\nabla }P_{j}=q_{j} n_{j}({\textbf{E}}+{\textbf{v}}_{j}\times {\textbf{B}})+\frac{\hbar ^{2}}{4m_{j} }\nabla \mathbf {(}\nabla ^{2}n_{j})-\frac{2n_{j0}\mu _{j}}{\hbar }\nabla (\mathbf {S\cdot B}_{1}) \end{aligned}$$

where $n_{j}$ is the particles number density of *jth* $(=i,e,p)$ particle which allow us to write the quasi-neutrality condition as $n_{i0} =n_{e0}-n_{p0}$. $( \rho =mn)$ is the particle density, ${\textbf{v}}_{j}$ and $P_{j}$ is the fluid velocity and thermal pressure, respectively. $q_{j}$, ${\textbf{E}}$ and ${\textbf{B}}$ are the electric charge, electric field and magnetic field, respectively. For the degenerate electrons and positron we use Fermi pressure as $P_{Fj}=\frac{(3\pi ^{2})^{\frac{2}{3} }\hbar ^{2}}{5m_{j}}n_{j}^{\frac{5}{3}}$ and $\nabla P_{Fj}=\frac{1}{3} v_{Fj}^{2}m_{j}\nabla n_{j}$ with $v_{Fj}=(3\pi ^{2}n_{j0})^{\frac{1}{3}} \frac{\hbar }{m_{j}}$ representing the Fermi velocity and for the massive non-degenerate ions, one can use the classical pressure as $P_{i}=\gamma _{i}n_{i}k_{B}T_{i}$ with $\gamma _{i}$ is the polytropic index. On the left-hand side of the equation, we have the continual derivative of the velocity field $\frac{d_{j}}{dt}=\frac{\partial }{\partial t}+{\textbf{v}} _{j}\cdot \mathbf {\nabla }$ and the gradient of pressure. On the right-hand side of the Euler equation, we present the force fields of different natures. The first term is the Lorentz force term, The second term represents the quantum Bohm potential. The last term on the right hand side represents the effect of spin magnetization force. The parameter $\mu _{j}=\frac{q\hbar }{2m_{j}c}$ represents the magnetic moment of *jth* particle and $B_{1}$ stands for the perturbed magnetic field. We can define the electron magnetic moment as $\mu _{e}=-\mu _{B}$, with $\mu _{B}=\mid \frac{q\hbar }{2m_{j}c}\mid$ being the Bohr magneton. $\hbar$ being the reduced plank’s constant. The spin evolution equation for the spin-1/2 quantum plasma can be written as $\frac{ds}{dt}=\frac{2\mu }{\hbar }({\textbf{s}}\times {\textbf{B}})$. Under the MHD limitations $\left( \omega \le \omega _{ci}\le \omega _{ce}\right)$, the spin inertia can be neglected well below the electron cyclotron frequencies, gives the spin equation of motion with solution^26^

3 $$\begin{aligned} {\textbf{S}}=-\frac{\hbar }{2}\eta (\frac{\mu _{j}{\textbf{B}}}{k_{B}T_{Fj}}){\hat{B}} \end{aligned}$$

Here $\left( \eta (\alpha _{j})=\tanh (\alpha _{j})\right)$ is the Langevin parameter with $\alpha _{j}=\frac{\mu _{B}B_{0}}{k_{B}T_{Fj}}$, and $T_{Fj}=\frac{(3\pi ^{2}n_{j})^{2/3}\hslash ^{2}}{2k_{B}m_{j}}$ is the degenerate Fermi temperature of the $j_{th}$ species. The above set of continuity and momentum equations are coupled to maxwell’s equations in the form

4 $$\begin{aligned}{} & {} \mathbf {\nabla }\cdot {\textbf{E}}=\frac{e}{\epsilon _{0}}(n_{i}-n_{e}-n_{p}), \end{aligned}$$

5 $$\begin{aligned}{} & {} \mathbf {\nabla }\cdot {\textbf{B}}=0, \end{aligned}$$

6 $$\begin{aligned}{} & {} \partial _{t}{\textbf{B}}=-\mathbf {\nabla }\times {\textbf{E}} \end{aligned}$$

and

7 $$\begin{aligned} \mathbf {\nabla }\times {\textbf{B}}=\mu _{0}{\textbf{J}}+\frac{1}{c^{2}}\partial _{t}{\textbf{E}} \end{aligned}$$

where ${\textbf{J}}=\sum _{j=e,i}q_{j}n_{j}v_{j}+cJ_{Me}+cJ_{Mp}$ is the current density with $J_{Me}=\mathbf {\nabla }\times {\textbf{M}}_{e}$ and $J_{Mp} =\mathbf {\nabla }\times {\textbf{M}}_{p}$ being the spin magnetization current densities of electrons and positrons, respectively. The magnetization density vector is $\mathbf {Me=}\mu _{B}n_{e}\tanh (\alpha ){\hat{B}}$ and $c=(\varepsilon _{0}\mu _{0})^{-\frac{1}{2}}$ is the speed of light in vacuum.

In a cylindrical coordinates system, the perturbed magnetic and electric fields are ${\textbf{B}}_{1}=({\widetilde{B}}_{r},{\widetilde{B}}_{\theta },{\widetilde{B}} _{z})$ and ${\textbf{E}}_{1}=({\widetilde{E}}_{r},{\tilde{E}}_{\theta } ,{\widetilde{E}}_{z}),$ and velocity ${\textbf{v}}_{j1}=({\widetilde{v}} _{jr},{\widetilde{v}}_{j\theta },{\widetilde{v}}_{jz})$. While ${\widetilde{P}}_{j}$ and ${\widetilde{n}}_{j}$ are the perturbed pressure and perturbed number density, respectively. Each perturbed profile is considered to be proportional to $e^{-i\omega t+ik_{z}z}$, where $\omega$ is the wave frequency and $k_{z}$ is the wave number directed along *z*-axis. Due to the incompressibility the mass conservation is reduced to $\mathbf {\nabla }\cdot {\textbf{v}}_{j}=0$, gives rise to ${\widetilde{v}}_{jz}=i\frac{{\hat{L}}}{k_{z}}{\widetilde{v}}_{jr}.$ The perturbed Poisson’s equation $\nabla \cdot {\textbf{E}}_{1}=0$, resulting in ${\hat{E}}_{z}=i\frac{{\hat{L}}}{k_{z}}{\tilde{E}}_{r}$. Form the divergence free property of the magnetic field $\nabla \cdot {\textbf{B}}_{1}=0$ gives rise to ${\textbf{B}}_{z}=i\frac{{\hat{L}}}{k_{z}}{\widetilde{B}}_{r}$ and from the perturb Faraday’s law we can get ${\hat{E}}_{\theta }=\frac{\omega }{k_{z}}{\widetilde{B}} _{r}$ and ${\textbf{B}}_{\theta }=\dfrac{k_{z}^{2}-\partial _{r}{\hat{L}}}{\omega k_{z}}{\tilde{E}}_{r}$. Here we define the operator ${\hat{L}}=\frac{1}{r}+\partial _{r}$. For instance neglecting the contribution of quantum Bohm potential in the momentum equation and only incorporating the contribution from spin magnetization, the linearized equation (2) in component $(r,\theta ,z)$ form can be expressed as

8 $$\begin{aligned}{} & {} \frac{\partial }{\partial t}{\tilde{v}}_{jr}{\hat{r}}-2\Omega {\tilde{v}}_{j\theta }{\hat{r}}=-\frac{\nabla _{r}}{m_{j}n_{j}}{\tilde{P}}_{e}+\frac{q_{j}}{m_{j} }[{\widetilde{E}}_{r}+{\tilde{v}}_{0}{\tilde{B}}_{z}+{\tilde{v}}_{\theta }{\tilde{B}} _{0}]{\hat{r}}\text {,} \end{aligned}$$

9 $$\begin{aligned}{} & {} \frac{\partial }{\partial t}{\tilde{v}}_{j\theta }{\hat{\theta }}+\frac{\kappa ^{2} }{2\Omega }{\tilde{v}}_{jr}{\hat{\theta }}=-\frac{\nabla _{\theta }}{m_{j}n_{j}} {\tilde{P}}_{j}+\frac{q_{j}}{m_{j}}[{\tilde{E}}_{\theta }-{\tilde{v}}_{r}\tilde{B}_{0}]{\hat{\theta }}\text {,} \end{aligned}$$

and

10 $$\begin{aligned} \frac{\partial }{\partial t}{\tilde{v}}_{jz}{\hat{z}}=-\frac{\nabla _{z}}{m_{j} n_{j}}{\tilde{P}}_{j}+\frac{q_{j}}{m_{j}}[{\tilde{E}}_{z}-{\tilde{v}}_{0}\tilde{B}_{r}]{\hat{z}}-\eta _{j}(\alpha )\mu _{j}n_{j}\nabla _{z}B_{z}\text {.} \end{aligned}$$

where $\kappa ^{2}=\frac{d\Omega ^{2}}{d\ln r}+4\Omega ^{2}$ is the square of the epicyclic frequency. Applying the space and time Fourier transform on the above Eqs. (8), (9) and (10) we can write the corresponding electron, positron and ion equations of motion as

11 $$\begin{aligned}{} & {} -i\omega {\widetilde{v}}_{er}-\left( 2\Omega -\omega _{ce}\right) {\tilde{v}}_{e\theta }=\frac{-i\omega }{k_{z}^{2}}\partial _{r}{\hat{L}}{\tilde{v}} _{er}+\frac{\omega \omega _{ce}}{k_{z}B_{0}}B_{\theta }-\frac{i}{k_{z}}\left( \frac{\omega _{ce}}{B_{0}}\frac{d\Omega }{d\ln r}+\eta _{e}(\alpha )\mu _{e} n_{e0}\partial _{r}{\hat{L}}\right) {\tilde{B}}_{r} \end{aligned}$$

12 $$\begin{aligned}{} & {} -i\omega {\widetilde{v}}_{e\theta }+(\frac{\kappa ^{2}}{2\Omega }-\omega _{ce} ){\tilde{v}}_{er}=\frac{\omega \omega _{ce}}{k_{z}B_{0}}B_{r}\text {,} \end{aligned}$$

13 $$\begin{aligned}{} & {} -i\omega {\widetilde{v}}_{pr}-\left( 2\Omega +\omega _{cp}\right) \tilde{v}_{p\theta }=\frac{-i\omega }{k_{z}^{2}}\partial _{r}{\hat{L}}{\tilde{v}} _{pr}+\frac{\omega \omega _{cp}}{k_{z}B_{0}}B_{\theta }-\frac{i}{k_{z}}\left( \frac{\omega _{cp}}{B_{0}}\frac{d\Omega }{d\ln r}+\eta _{p}(\alpha )\mu _{e} n_{p0}\partial _{r}{\hat{L}}\right) {\tilde{B}}_{r} \end{aligned}$$

14 $$\begin{aligned}{} & {} -i\omega {\widetilde{v}}_{p\theta }+(\frac{\kappa ^{2}}{2\Omega }+\omega _{cp} ){\tilde{v}}_{pr}=-\frac{\omega \omega _{cp}}{k_{z}B_{0}}B_{r} \end{aligned}$$

and

15 $$\begin{aligned}{} & {} -i\omega {\widetilde{v}}_{ir}-\left( 2\Omega +\omega _{ci}\right) {\tilde{v}}_{e\theta }=\frac{-i\omega }{k_{z}^{2}}\partial _{r}{\hat{L}}{\tilde{v}} _{ir}+\frac{\omega \omega _{ci}}{k_{z}B_{0}}B_{\theta }-\frac{i\omega _{ci}}{k_{z}B_{0}}\frac{d\Omega }{d\ln r}{\tilde{B}}_{r} \end{aligned}$$

16 $$\begin{aligned}{} & {} -i\omega {\widetilde{v}}_{i\theta }+(\frac{\kappa ^{2}}{2\Omega }+\omega _{ci} ){\tilde{v}}_{ir}=-\frac{\omega \omega _{ci}}{k_{z}B_{0}}B_{r}\text {.} \end{aligned}$$

The quantum contributions to the momentum equation associated with the ions have been neglected because of their heavier mass in comparison to the electron and positron. Here $\omega _{ce}=\frac{eB_{0}}{m_{e}}$ is the electron cyclotron frequency associated with external magnetic field, $\Omega _{c} =\frac{eB_{0}}{m_{i}}$ stands for ion gyrofrequency and $\Omega _{p} =\frac{eB_{0}}{m_{p}}$ stands for positron gyrofrequency and $\omega$ is the wave frequency. The local approximations are adopted, assuming $\partial _{r}\simeq ik_{r}$ and $k_{r}r\gg 1,$ where $k_{r\text { }}$is the radial wave number. Thus $\partial _{r}{\hat{L}}\simeq -k_{r}^{2}$ and $k=(k_{r}^{2}+k_{z} ^{2})^{1/2}$ is the total wave number. The perturbed magnetic field can be determined by using

17 $$\begin{aligned} \nabla \times {\textbf{B}}_{1}=en_{0}\mu _{0}({\textbf{v}}_{i1}-{\textbf{v}} _{e1}+{\textbf{v}}_{p1})+S_{t}\left( \mathbf {\nabla }\times {\textbf{B}} _{1}\right) \end{aligned}$$

where $S_{t}=\left( S_{e}-S_{p}\right)$ with $S_{e}=\eta _{e}(\alpha )\mu _{e}n_{e}$ and $S_{p}=-\eta _{p}(\alpha )\mu _{p}n_{p}$. By using the problem geometry the ion perturbed velocities ${\tilde{v}}_{ir}$ and ${\tilde{v}} _{i\theta }$ are obtained from Eq. (17) as

18 $$\begin{aligned}{} & {} {\tilde{v}}_{ir}=-\frac{ik_{z}}{en_{0}\mu _{0}}{\tilde{B}}_{\theta }-\frac{ik_{z}^{2}}{en_{0}\mu _{0}}S_{t}{\tilde{B}}_{\theta }+{\tilde{v}}_{er}-\tilde{v}_{pr} \end{aligned}$$

19 $$\begin{aligned}{} & {} {\tilde{v}}_{i\theta }=\frac{ik^{2}}{k_{z}en_{0}\mu _{0}}{\tilde{B}}_{r} +\frac{ik^{2}}{en_{0}\mu _{0}}S_{t}{\tilde{B}}_{r}+{\tilde{v}}_{e\theta }-\tilde{v}_{p\theta }. \end{aligned}$$

From $\theta$-component of the positron equation of motion, we can find the positron velocities ${\tilde{v}}_{pr}$ and ${\tilde{v}}_{p\theta }$ as

20 $$\begin{aligned} {\tilde{v}}_{pr}=\frac{1}{D_{1}}\left[ \left\{ \frac{i\Omega _{p}}{k_{z}B_{0}} (\frac{\kappa ^{2}}{2\Omega }-\Omega _{p})+\frac{k^{2}}{k_{z}^{2}}\eta _{p}(\alpha )\mu _{p}n_{p}\right\} {\tilde{B}}_{r}+\frac{\omega \Omega _{p}}{k_{z}B_{0}}{\tilde{B}}_{\theta }\right] \end{aligned}$$

and

21 $$\begin{aligned} {\tilde{v}}_{p\theta }=\frac{\Omega _{p}}{D_{1}k_{z}B_{0}}\left[ \omega \frac{k^{2}}{k_{z}^{2}}\left\{ 1+\frac{k_{z}^{2}}{\omega ^{2}k^{2}} (\frac{\kappa ^{2}}{2\Omega }-\Omega _{p})\frac{d\Omega }{d\ln r}-\eta _{p} (\alpha )\mu _{p}n_{p}\right\} {\tilde{B}}_{r}+i(\frac{\kappa ^{2}}{2\Omega }-\Omega _{p})\right] \end{aligned}$$

The dispersion relation corresponding to Eqs. (11–16) and (18–21) can be obtained as

22 $$\begin{aligned} \left. \begin{array}{c} \left( \omega ^{2}\alpha _{0}+\omega _{A}^{2}\beta \right) ^{2}-\frac{\omega ^{2}\Omega _{p}^{2}}{D_{1}^{2}\omega _{ce}^{2}}D_0\left[ \omega \frac{k^{2}}{k_{z}^{2}}\left\{ (\frac{\kappa ^{2}}{2\Omega }-\omega _{ce})-(\frac{\kappa ^{2}}{2\Omega }-\Omega _{p})+\left( 2\Omega +\Omega _{c}\right) \right\} +\frac{k^{2}}{k_{z}^{2}}\left\{ \left( 2\Omega +\Omega _{c}\right) S_{t}\left( \frac{1}{\omega }-\Omega _{p}\right) \right\} \right. \\ \left. -\frac{1}{\omega }\left\{ \frac{k^{2}}{k_{z}^{2}}S_{t}+(\frac{\kappa ^{2}}{2\Omega }-\Omega _{p})^{2}\left( 2\Omega +\Omega _{c}\right) \right\} \right] +\omega \frac{k^{2}}{k_{z}^{2}}\left[ \frac{k^{2}V_{A}^{2} }{\Omega _{c}\omega _{ce}}\left\{ (\frac{\kappa ^{2}}{2\Omega }-\omega _{ce})+\left( 2\Omega +\Omega _{c}\right) \left( \frac{k^{2}V_{A}^{2}}{\Omega _{c}\omega _{ce}}-1\right) \right. \right. \\ \left. \left. -(\frac{\kappa ^{2}}{2\Omega }-\omega _{ce})+S_{p}+\left( 2\Omega +\Omega _{c}\right) S_{t}\right\} \right] \times \left[ \frac{1}{\omega }\left( \frac{k^{2}V_{A}^{2}}{\Omega _{c}}\right) +(\frac{\kappa ^{2}}{2\Omega }-\Omega _{c})\left( 1-\frac{k^{2}V_{A}^{2}}{\Omega _{c}\omega _{ce}}\right) +\frac{\omega _{A}^{2}}{\omega ^{2}}\beta \frac{d\Omega }{d\ln r}(1+S_{t})+\right. \\ \left. \left\{ \frac{\kappa ^{2}}{2\Omega }\left( \alpha _{0}-1-\frac{\Omega _{c}}{\omega _{ce}}\right) -\frac{k^{2}V_{A}^{2}}{\Omega _{c}}\left( 1-\frac{\Omega _{c}}{\omega _{ce}}\right) -\frac{\omega _{A}^{2}}{\omega ^{2} }\beta \frac{d\Omega }{d\ln r}\right\} S_{t}\right] +\frac{\omega ^{2}}{D_{1}^{2}\omega _{ce}}\left[ \omega \left\{ (\frac{\kappa ^{2}}{2\Omega }-\omega _{ce})-(\frac{\kappa ^{2}}{2\Omega }-\Omega _{p})+\left( 2\Omega +\Omega _{c}\right) \right\} \right. \\ \left. +\left\{ \left( 2\Omega +\Omega _{c}\right) \left( \frac{1}{\omega }-\Omega _{p}\right) S_{t}+\frac{S_{p}}{\omega }\right\} -\frac{1}{\omega _{ce}}\left\{ S_{t}+(\frac{\kappa ^{2}}{2\Omega }-\Omega _{p})(\frac{\kappa ^{2} }{2\Omega }-\omega _{ce})\left( 2\Omega +\Omega _{c}\right) \right\} \right] =0 \end{array} \right. \end{aligned}$$

where $V_{A}=\sqrt{\dfrac{B_{0}^{2}}{m_{i}n_{i}\mu _{0}}}$ and $\omega _{A}=k_{z}V_{A}$ are the Alfvén speed and frequency, respectively. Here we denote

$$\begin{aligned}{} & {} \alpha _{0}=1+\frac{k^{2}V_{A}^{2}}{\Omega _{c}\omega _{ce}}+\frac{\Omega _{c}}{\omega _{ce}}\text { \ and \ }\beta =\frac{1}{\Omega _{c}\omega _{ce}}\left( \frac{\kappa ^{2}}{2\Omega }-\omega _{ce}\right) \left( \frac{\kappa ^{2}}{2\Omega }+\Omega _{c}\right) \\{} & {} D_{1}=-i\omega \frac{k^{2}}{k_{z}^{2}}+\frac{i}{\omega }\left( \frac{\kappa ^{2}}{2\Omega }-\Omega _{p}\right) \left( 2\Omega +\Omega _{p}\right) \end{aligned}$$

The formula refers in Eq. (22) is of complex nature and complicated to investigate analytically in the present form. It contains the information about MRI in high, intermediate and low frequency regimes.

## Reduced dispersion relation

Equation (22) reveals the contribution of spin magnetization force to the dispersion of wave depending on the magnetic field *B* strength and orientation of the electron and positron. The obtained DR (22) for the given multi-species (e–p–i) rotating plasma system is too complicated to analyze directly. To understand the spin contribution of both the plasma ingredients (electron and positron) we limit ourselves to the low frequency or longer wavelength MHD approximations i.e. $kV_{A}\ll \Omega _{c}$, $\omega \ll \Omega _{c}$, $\Omega \ll \Omega _{c}$ assuming $\frac{\Omega _{c}}{\omega _{ce} }=\frac{m_{e}}{m_{i}}\simeq 0,$ $\alpha _{0}\simeq 1,$ and $\beta \simeq -1$, the DR (22) can be expressed as

23 $$\begin{aligned} \omega ^{2}-k_{z}^{2}V_{A}^{2}-\frac{k_{z}^{2}}{k^{2}}\left[ \frac{4\Omega ^{2}\omega ^{2}}{\omega ^{2}-k_{z}^{2}V_{A}^{2}}+\frac{d\Omega ^{2}}{d\ln r}-\frac{k_{z}^{2}V_{A}^{2}}{\omega ^{2}-k_{z}^{2}V_{A}^{2}}\frac{d\Omega }{d\ln r}S_{t}\right] =0 \end{aligned}$$

Equation (23) is the reduced dispersion relation for the MRI in three component ideal MHD model with the effect of spin magnetization correction. If the effect of spin magnetization is set to be zero ($S_{t}=0$) in Eq. (23), the classical dispersion relation for two fluid model recovers^63^. In some magnetized plasmas, the contribution of spin magnetization is small to the total magnetic field. In a low temperature and high density plasmas like that in the locality of magnetars and pulsars, the contributions appears due to the fact that the component of spin force is in line to the ambient magnetic field. For the higher values of magnetic field *B* the magnetization energy shows some important consequences on the dynamics of the system. To demonstrate the instability, we can write the DR (22) in the form

24 $$\begin{aligned} \omega ^{4}\alpha ^{2}+\omega ^{2}\frac{k_{z}^{2}}{k^{2}}D_{2}-\frac{k_{z}^{4} }{k^{4}}\left[ k^{4}V_{A}^{4}+k^{2}V_{A}^{2}\frac{d\Omega }{d\ln r}\right] D_{5}=0\text {,} \end{aligned}$$

Here $D_{2}=D_{0}\left( D_{3}-D_{4}\right)$ and $D_{5}=D_{3}-D_{4}$. Where $D_{0}$, $D_{3}$ and $D_{4}$ are as following

$$\begin{aligned}{} & {} D_{0}=\left[ \frac{\kappa ^{2}}{2\Omega }\left( 2\alpha _{0}-1-\frac{\Omega _{c}}{\omega _{c}}\right) -\frac{k^{2}V_{A}^{2}}{\Omega _{c}}\left( 1-\frac{\Omega _{c}}{\omega _{c}}\right) \right] \text {,}\\{} & {} D_{3}=\left[ 2\Omega \alpha _{0}+\frac{\kappa ^{2}}{2\Omega }\left( \alpha _{0}-1-\frac{\Omega _{c}}{\omega _{c}}\right) -\frac{k^{2}V_{A}^{2}}{\Omega _{c}}\left( 1-\frac{\Omega _{c}}{\omega _{c}}\right) \right] \end{aligned}$$

and

$$\begin{aligned} D_{4}=\frac{k^{2}V_{A}^{2}}{\Omega _{c}}\left[ \frac{1}{\omega _{c}}\left( \frac{\kappa ^{2}}{2\Omega }-\omega _{c}\right) -\left( 1-\frac{\Omega _{c} }{\omega _{c}}\right) -\omega _{A}^{2}\frac{d\Omega }{d\ln r}S_{t}\right] -2\alpha _{0}k^{2}V_{A}^{2}\beta \end{aligned}$$

Equation can be expressed in the form of

25 $$\begin{aligned} \omega ^{4}\alpha _{0}^{2}-\omega ^{2}\left( 2\omega _{A}^{2}+\frac{k_{z}^{2} }{k^{2}}\kappa ^{2}\alpha _{0}(1+S_{t})\right) +\omega _{A}^{2}\left( \frac{\omega _{A}^{2}}{\alpha _{0}}+\frac{k_{z}^{2}}{k^{2}}\frac{d\Omega ^{2} }{d\ln r}S_{t}\right) \end{aligned}$$

Equation (25) is a biquadratic equation in $\omega$. The MRI growth rate $\left( \gamma =-i\omega \right)$ can be determined by using the following relation

26 $$\begin{aligned} \gamma ^{2}=\frac{\sqrt{\Delta }-b_{0}}{2b} \end{aligned}$$

where $$b=\alpha _{0}^{2}$$, $$b_{0}=2\omega _{A}^{2}+\frac{k_{z}^{2}}{k^{2}} \kappa ^{2}\alpha _{0}(1+S_{t})$$ and

$$\begin{aligned} \Delta =\frac{4\omega _{A}^{2}\kappa ^{4}\alpha _{0}\delta S_{t}}{1+S_{t}} +\kappa ^{2}\delta ^{2}\alpha _{0}\left\{ 4\omega _{A}^{2}+\kappa ^{2}\delta ^{2}\left( 1+S_{t}\right) \right\} \end{aligned}$$

Here $\delta =\frac{k_{z}}{k}.$ We obtained a biquadratic equation given in Eq. (25) describing four different MR modes. Indeed, some of the modes can be unstable in some different conditions, but here in our current work we have studied only one purely unstable mode, giving the growth rate of MRI expressed in Eq. (26).

## Results and discussion

To probe the impact of different plasma parameters like magnetic field *B*, particle density *n* (electrons and positrons) and spin magnetization $$\eta$$, we evaluate Eq. (26) numerically to investigate the growth rate of MR modes. For this purpose, we have taken some typical degenerate plasma parameters related to some compact objects e.g. white dwarf and Neutron stars^13, 83–85^, the particle number densities are in the range $$( \sim 10^{30})$$ m$$^{-3}$$, magnetic field *B* strength is of the order $$\sim$$ megatesla to teratesla and the temperatures lie in the range $$( 10^{5}$$–$$10^{7})$$ K.

**Figure 1:**
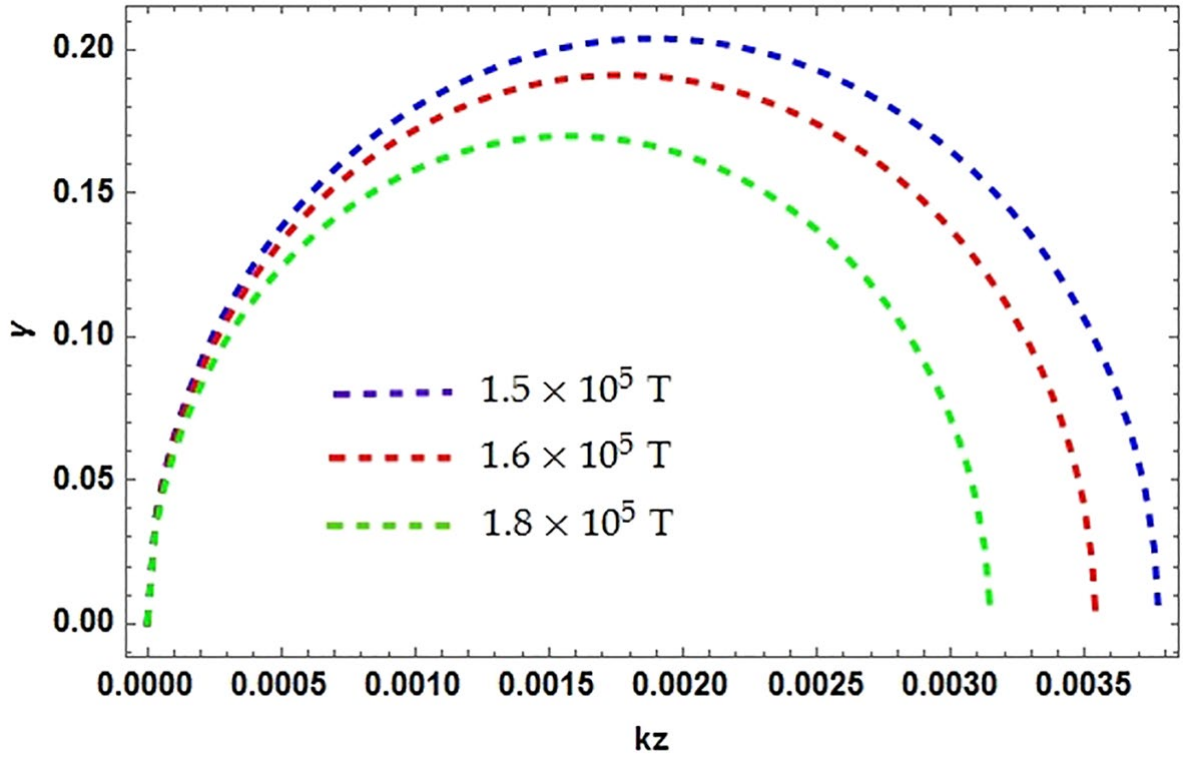
The normalized growth growth rate $\gamma$ over the wave vector $k_{z}.$ assuming $\Omega =10^{3}$ and $\kappa ^{2}<0$, all othe typical parameters are $n_{e0}=10^{31}$, $n_{p0}=10^{30}$, $\alpha _{0}\simeq 1,$ and $\beta \simeq -1$. Varying only the value of magnetic field *B* as dotted blue curve $B=1.5\times 10^{5}$ T, dotted red curve $B=1.6\times 10^{5}$ T and dotted green $B=1.8\times 10^{5}$ T.

We plotted the dependence of growth rate of unstable MR mode $\gamma$ against wave vector $k_{z}$ in Fig. 1 to study the effect of the background magnetic field *B*. We have obtained two curves (blue $1.5\times 10^{5}$ T) and (red $1.2\times 10^{5}$ T) for different magnetic field strength in the presence of the magnetization effect $\eta$ from both electron and positron. It is clearly shown that the magnetic field enforce the growth rate of the mode towards stability. The spin terms are of particular significance and importance for low temperature and strong magnetized plasmas, when the spin are aligned with the field. We here in this work stress that the spin term can have more influence than the other terms in MHD equation. As a consequence it turns out that the spin force can be important even when the magnitude of the imposed magnetic field is smaller than the usual $J\times B$ force. In order to demonstrate this property, we have studied the growth rate of MR instability, when the strength of magnetic filed *B* increases the growth rate can become stable for some of the possible orientations of $k_{z}$. MRI take place in a weak magnetic field regions and enhancing the field by producing field amplification. The field will grow because of the MRI dynamo action until it reaches a saturation field limit. The details of the dynamo action is beyond the scope of this work. Strong magnetization effect is observed from both the ingredients of plasma where the field strength is higher, consequently impose a stability on the system. This instability always occurs in the vicinity of a low magnetic field where the toroidal field components dominate. This leading to the rapid growth of the *B* field whose characteristic time scale is of the order of fluid rotational period. The instability broadly occurs in the core collapse which is consider to be the dominant mechanism of magnetic flux production, has the capacity to strong enough to affect. If the field is not strong enough it cause the explosion in massive stars. Our calculations clearly presenting, that only a low or weak magnetic field *B* can affects the stability properties of the system. The spin effects become noticeable even when the external magnetic field $B_{{\textbf{0}}}$ is below the quantum critical magnetic field strength $(\sim 10^{10})$ T.

**Figure 2:**
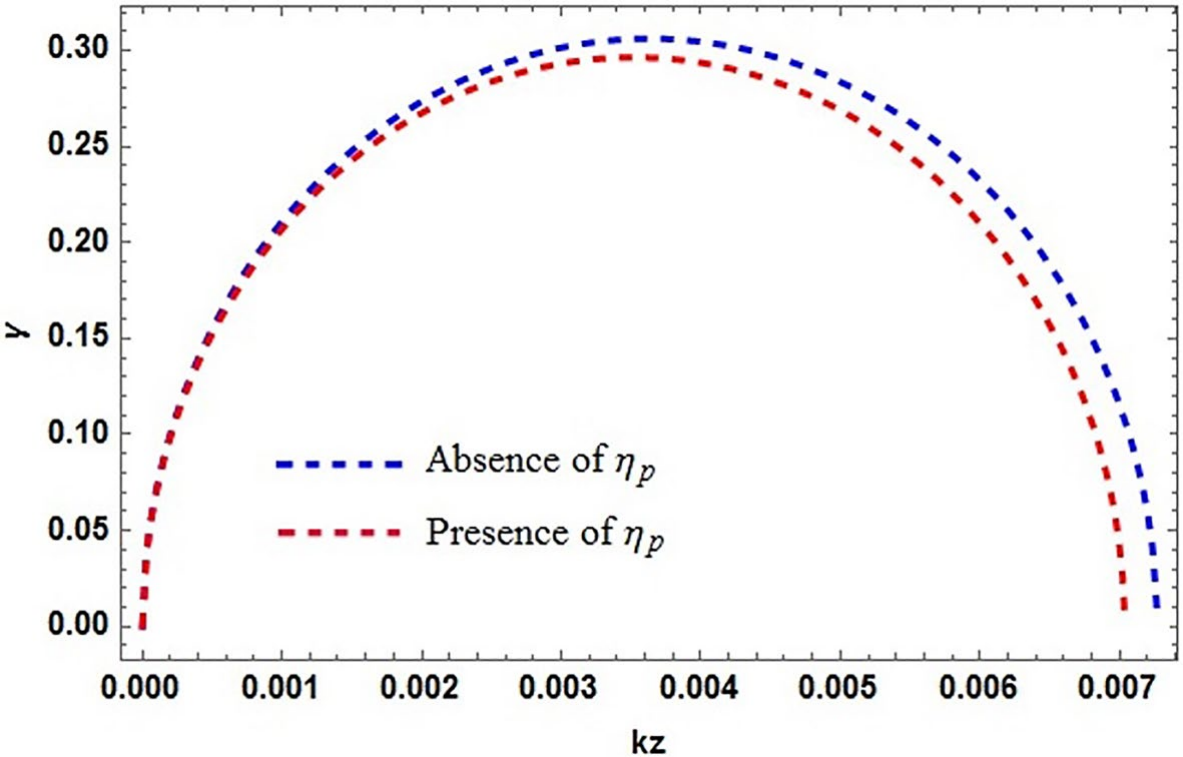
The normalized growth rate $\gamma$ versus wave vector $k_{z}$. The typical value of $\Omega =10^{3}$ and $\kappa ^{2}<0$, with electron and positron densities given in Fig. 1 and $\alpha _{0}\simeq 1,$ and $\beta \simeq -1$. Neglecting the contribution of positron spin magnetization effect $\eta _{p}=0$ (dotted blue curve), showing the increasing trend of growth rate $\gamma$. The stability is observed when the spin magnetization of both the species are taken into effect. Dotted red curve show the combine effect of both electron and positron magnetization.

**Figure 3:**
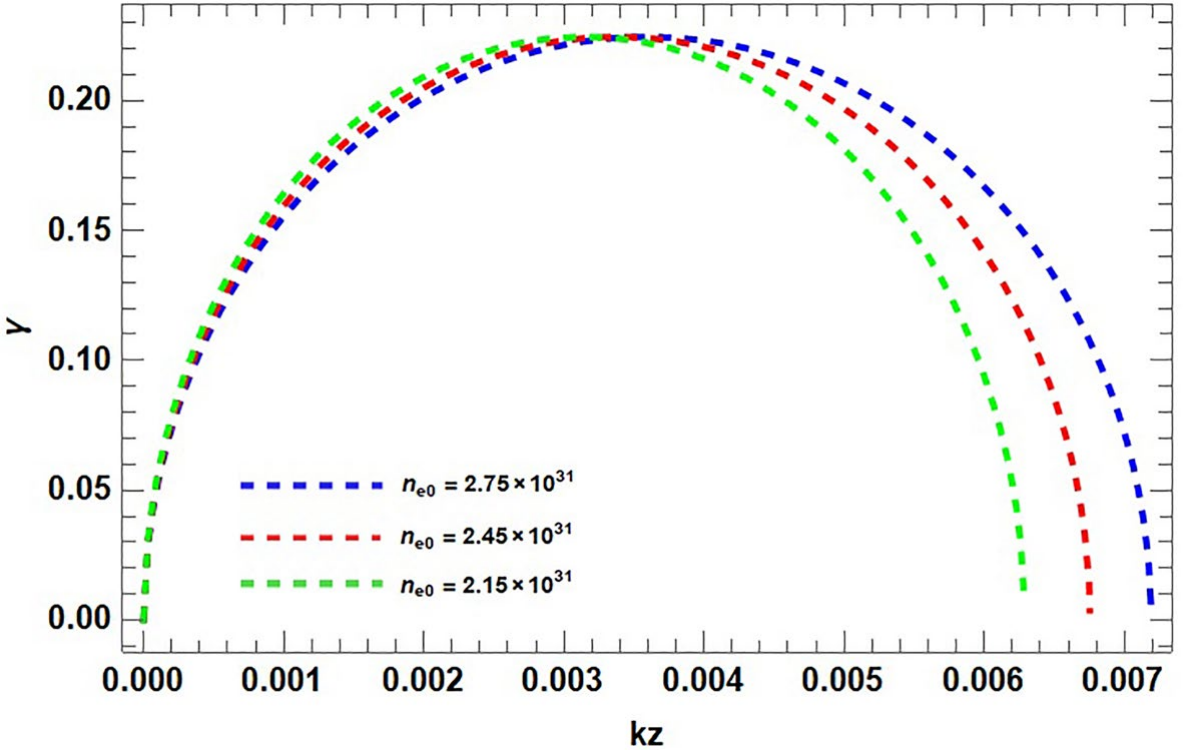
The normalized growth rate $\gamma$ is plotted against wave vector $k_{z}$ for various values of electron number density $n_{e0}$ . Assuming the same typical parameters, $\Omega =10^{3}$ and $\kappa ^{2}<0$, with fixed positron density $n_{p0}=0.2\times 10^{31}$. The dotted curve (blue) $n_{e0}=2.75\times 10^{31}$. The variation in the curve (red $n_{e0}=2.45\times 10^{31}$) and (green $n_{e0}=2.15\times 10^{31}$) is observed as $\gamma$ increase with the increasing value of $n_{e0}$.

**Figure 4:**
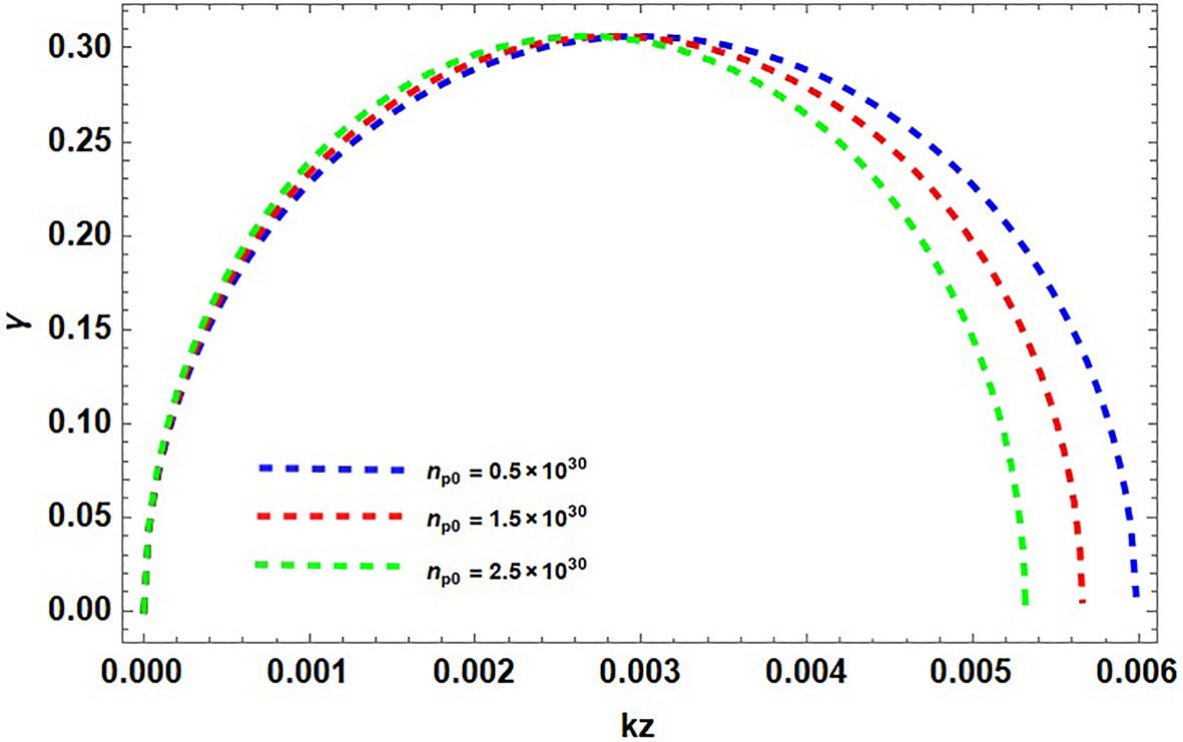
The plot shows the normalized growth rate $\gamma$ against wave vector $k_{z}$ for various values of positron number density $n_{p0}$ . Assuming the same typical parameters, $\Omega =10^{3}$ and $\kappa ^{2}<0$, with fixed electron number density $n_{e0}=10^{30}$. The dotted curve (blue) $n_{p0} =1\times 10^{30}$. The variation in the red curve ($n_{p0}=1.5\times 10^{30}$) is observed as the growth rate $\gamma$ decrease with the increasing value of $n_{p0}$.

In Fig. 2, the growth rate $\gamma$ of the MR mode is plotted against the wavevector $k_{z}$ in the absence and in the presence of contribution of spin magnetization from positron. By considering $S_{p}=0$, enhances the growth rate and system is less stabilized and is more stabilized in the presence of the contribution from both electron and positron. It is clear from the figure that increasing positron concentration in dense astrophysical (e–p–i) plasmas impose stabilizing effects on the system. Increase in the electron number density $n_{e}$, the effect of spin magnetization and consequently the instability of the system enhances as shown in Fig. 3. The plot having three different curves (red, blue and green), representing the variation in density gradient which modifies significantly the instability growth rate. This result is the confirmation of the diamagnetic behavior of plasma. Stellar plasmas becomes degenerate at high densities soon after the evolved star leaves the main sequence of formation and the structure readjusts. Degeneracy is important in white dwarfs stars and also in the central region of evolved stars because of the large densities found there. This result reveal our previous finding that the increasing electron densities destabilize the system and the growth rate consequently increases, also addressing the fact that the particle (electron) degeneracy pressure exceeds the external imposing fields and can take to the collapse of massive objects. It is clear that the degenerate electron gas cannot support a star with mass larger than the Chandrasekhar mass ($1.4M_{\odot }$). Conversely the increase in the positron number densities put stabilizing effects on the system. It is shown in Fig. 4 that, the presence of light positive species, i.e., positrons, can significantly modify the instability growth rate.

## Conclusions

In this current work, we have examined MRI in three component (e–p–i) plasmas using QHD model in a differentially rotating magnetized degenerate plasma. The DR is obtained with the contribution of spin magnetization force from electron and positron. Spin contributions has a significant importance in a high density, low temperature and highly magnetized plasmas that can be found in WDs. The DR has a complex nature and have the informations in all the frequency ranges. To briefly understand the dynamics of the system we limited ourselves to the longer wavelength (low frequency) MHD limits, and the reduced dispersion relation is obtained. To analyze the growth rate $\gamma$ of the instability we numerically solved the reduced dispersion relation and by using various astrophysical plasma (WD) parameters we plotted the dependence of growth rate $\gamma$ to the wave vector $k_{z}$. We obtained four different plots for the growth rate to study the effect of different parameters like magnetic field *B*, magnetization effect $\eta$, electron number density $n_{e}$ and positron number densities $n_{p}$. We concluded that the magnetic field strength has stabilizing effects on the growth rate. As the instability always takes place in the vicinity of a weak magnetic field, amplifies the field strength by the action of magnetic dynamos and pinches the system against the run away from stability. The electron spin magnetization force and the increasing electron number density enhance the growth of the MR mode and the system run towards instability. At very high number densities corresponding to MR instability results in the core collapse in many stars. On the other hand, the positron number density putting a stabilizing effect on the system. Therefore, the contribution from electron and positron fluids validates our consideration of their quantum mechanical effects in this model. The results of our findings presented here can be of particular importance for multi-species dense astrophysical environments.

## Data Availability

All data generated or analysed during this study are included in this published article.
